# Pulmonary Adenocarcinoma Presenting as Acute Heart Failure and Hypereosinophilia

**DOI:** 10.7759/cureus.21441

**Published:** 2022-01-20

**Authors:** Diana Ferrão, Clara Silva, Jorge S Almeida

**Affiliations:** 1 Internal Medicine, Centro Hospitalar Universitário de São João, Porto, PRT; 2 Medicine, Faculdade de Medicina da Universidade do Porto, Porto, PRT

**Keywords:** acute myocardial infarction, lung abscess, lung cancer, acute heart failure, hypereosinophilia

## Abstract

Hypereosinophilia is a serum eosinophil count of over 1,500 eosinophils/µL. It is an uncommon laboratory finding, and it can be asymptomatic or associated with organ damage, in which case it is referred to as hypereosinophilic syndrome. It can be primary, when the expansion of eosinophils occurs in the setting of a hematological neoplasm, or secondary, when it is caused by an external stimulus, such as a parasitic infection or a solid neoplasm. We present a case of hypereosinophilia diagnosed in a patient presenting with acute heart failure initially attributed to coronary disease and alcohol consumption. Due to persisting eosinophilia with no apparent cause, eventually reaching more than 41,000 eosinophils/µL, a full-body scan was performed, showing the presence of a peri-hilar mass partially obstructing the left main bronchus and multiple lesions in the liver and thoracic vertebrae. The liver biopsy revealed metastatic non-small cell lung carcinoma. Around the time the biopsy was performed, the patient began complaining of new-onset chest paint, and the diagnoses of pulmonary embolism and later lung abscess were made, the reasons why he had no conditions to begin chemotherapy. The medical condition of the patient deteriorated in the next few days, and the patient died six months after the initial diagnosis of hypereosinophilia.

## Introduction

Eosinophilia is defined as more than 500 eosinophils/µL of peripheral blood and hypereosinophilia as more than 1,500 eosinophils/µL. When this excess of peripheral eosinophils is associated with organ dysfunction, we are in the presence of a hypereosinophilic syndrome. It is usually idiopathic, but it can be caused by infections, drugs, immunological disorders, and solid and hematological neoplasms. Most commonly, eosinophilia is mild, asymptomatic, and not related to an underlying disorder and does not require a specific therapeutic approach. In rare cases, it can be severe and affect virtually any organ, ranging from mild symptomatic impairment to life-threatening multisystem failure. In these cases, an etiology should be searched for and, if found, corrected to prevent further clinical deterioration [1,2]. We present a case of eosinophilia diagnosed after hospital admission for acute heart failure.

## Case presentation

**Figure 1:**
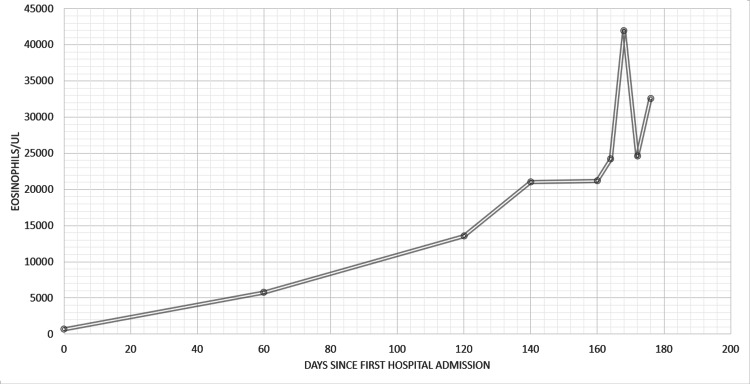
Eosinophil count since the first hospital admission The eosinophil count started normal and progressively increased since the first hospital admission for acute heart failure, reaching the impressive value of 40,000 eosinophils/uL; this high count is what motivated the search for an underlying diagnosis, despite the patient being totally asymptomatic.

We present the case of a 66-year-old male with no relevant medical history, except a smoking habit of 40 pack-years and sporadic alcohol consumption. He was brought to the emergency department (ER) because of sudden dyspnea in the middle of the night that woke him up. He denied having any degree of dyspnea before, affirming having only a mild productive cough that was usual due to his smoking habits. He also denied having chest pain, palpitations, dizziness, syncope, peripheral edema, or any other respiratory, gastrointestinal, or urinary complaints. At first medical contact in the ER, he was tachypneic with signs of respiratory distress. His blood pressure was 154/84 mmHg, and his heart rate was 110 beats/minute, with normal body temperature. Pulmonary auscultation revealed crackles in both lungs, from the base to the apex, with severe wheezing. He had no venous jugular turgescence or peripheral edema. An arterial blood gas (ABG) was performed, revealing type II respiratory failure with acidemia; he had hypoxemia with a pO_2_ of 56 mmHg, severe hypercapnia with a pCO_2_ of 75 mmHg, and respiratory acidemia, with pH 7,069. The electrocardiogram (ECG) showed occasional ventricular premature beats and new-onset ST-segment depression in inferior leads. The chest X-ray revealed the expected pattern of pulmonary congestion but was otherwise irrelevant. Complete blood count (CBC) and chemistry showed mild leukocytosis of 13,450 leukocytes/µL (reference range: 4,000-12,000 leukocytes/µL), mildly elevated liver enzymes, serum creatinine of 1.27 mg/dL (estimated glomerular filtration rate: 56 mL/minute), and normal C-reactive protein (CRP). Furthermore, it showed high-sensitivity cardiac troponin (hs-cTn) of 365 ng/L that rose to 7,493 ng/L three hours later (reference value: <34 ng/L) and brain natriuretic peptide (BNP) of 1,318 pg/mL (reference value: <100 pg/mL). The search for respiratory viruses (influenza and respiratory syncytial virus) was negative. The transthoracic echocardiogram displayed a severely compromised left ventricular function with global hypokinesis with a right ventricular function in the lower limit of normal. He was diagnosed with acute myocardial infarction Killip III and started on noninvasive ventilation and a high dosage of intravenous furosemide, with resolution of the pulmonary congestion and respiratory failure in 48 hours. He remained electrically and hemodynamically stable. The hs-cTn peaked at 19,000 ng/L, with a subsequent fall in the following few days. Cardiac catheterization was performed, showing 60% obstruction of the anterior descending artery and 60% of the circumflex artery, none of which were clearly the culprit lesion. Myocardial perfusion scintigraphy did not display a reversible component of these lesions, so a percutaneous coronary intervention was not attempted. The cardiac magnetic resonance imaging (cMRI) confirmed the global hypokinesis and showed myocardial late gadolinium enhancement in the heart septum, compatible with nonischemic cardiomyopathy. The etiology of the ventricular dysfunction was unclear but was interpreted as possible alcoholic cardiomyopathy with superimposed ischemic acute changes. The patient was discharged to the outpatient clinic. In the first appointment, he was asymptomatic and had resumed his normal life. However, a persistent rise in eosinophils was noted in his blood work. It had started during the time he spent in the infirmary, but since it was mild and not associated with an obvious cause, it was dismissed. However, it was progressively increasing since hospital discharge (Figure 1).

**Figure 2:**
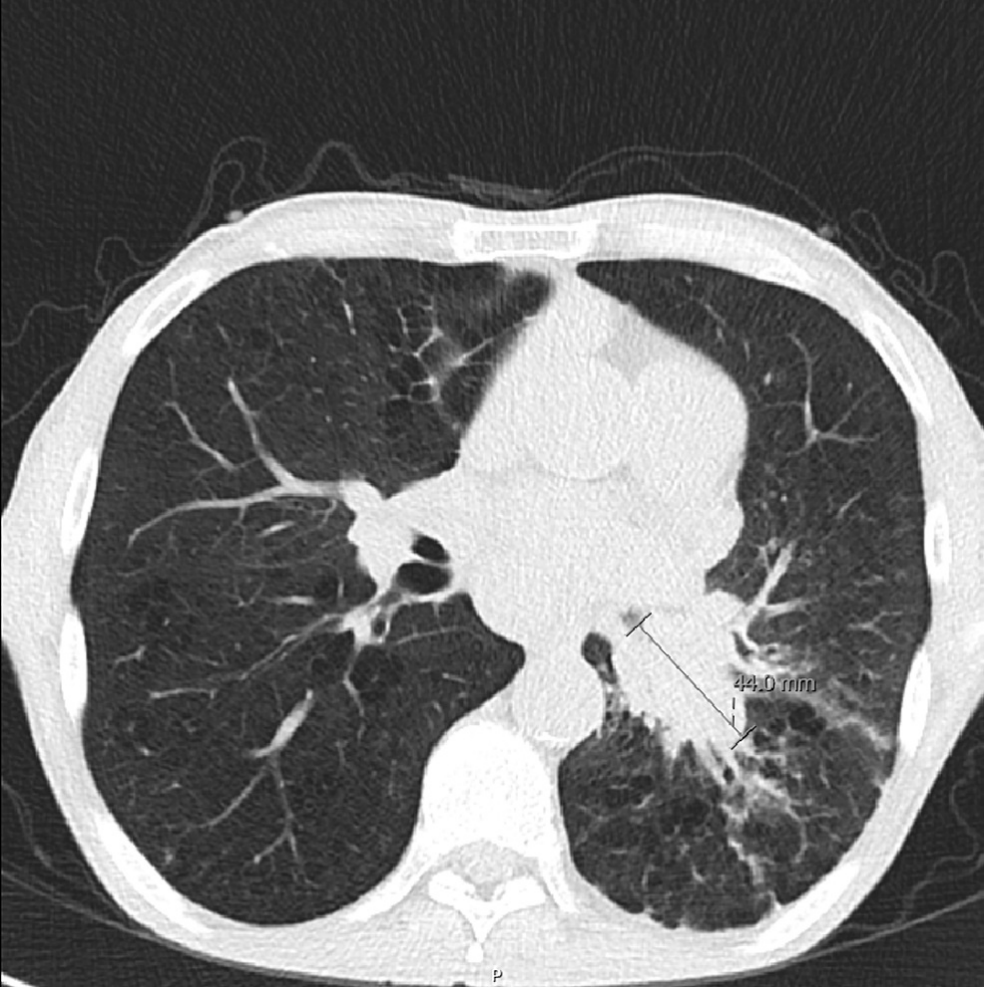
Lung peri-hilar mass obliterating the left main bronchus A large lung mass located in the left peri-hilar and peri-cardiac regions is apparent; it is spiculated and poorly defined, which, in association with the impressive hypereosinophilia that the patient presented with, made the diagnosis of lung malignancy likely.

**Figure 3:**
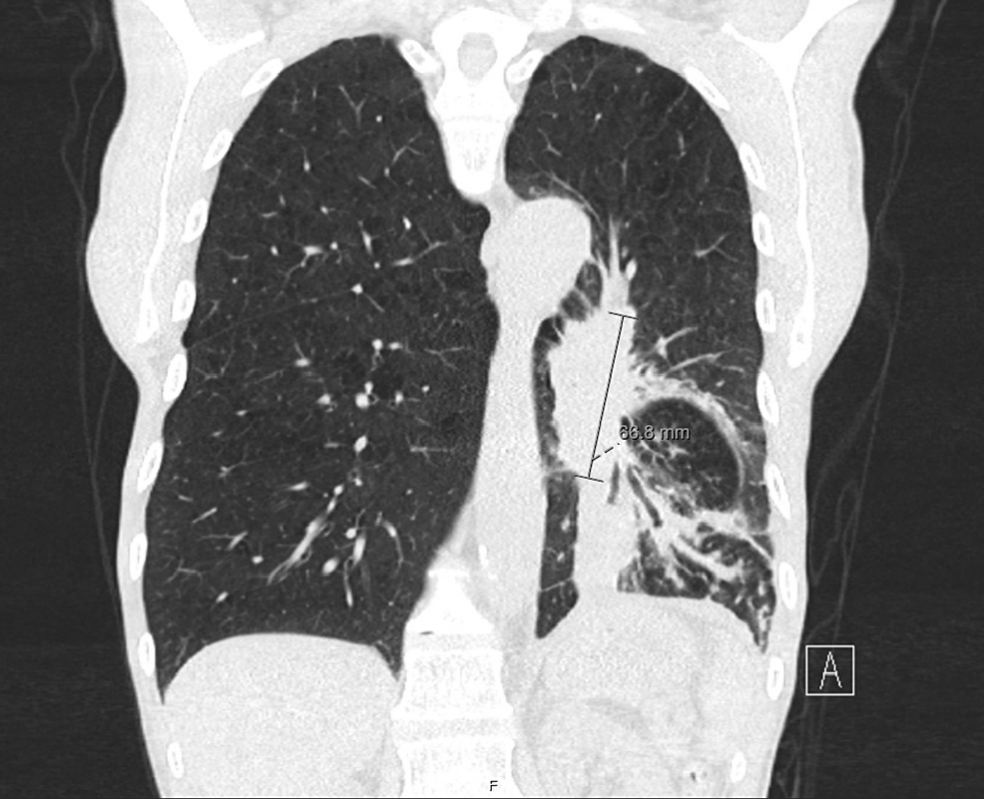
Lung peri-hilar mass hiding behind the cardiac shadow In the coronal view, we can see that the lung mass, albeit its important size, is largely hidden behind the cardiac silhouette, which justified why it was not apparent in the posteroanterior view of the X-ray.

Due to this fact, an etiology was actively investigated. He had never had asthma, allergic rhinitis, or any other signs of atopy. He had not started any new drugs, prescribed or off-the-counter, and no other clinical signs of drug hypersensitivity were found, such as cutaneous rash or joint pain. The absence of cutaneous and articular changes also made an inflammatory or autoimmune etiology less likely. The autoimmune panel showed a near-normal sedimentation rate of 30 mm/hour and a normal proteinogram and immunoglobulin panel, with the exception of a rise in IgE of 2,115 kU/L (reference value: <114 kU/L). The antinuclear antibodies were negative. He denied having fever or other complaints related to a possible infectious disease by parasites, viruses, or fungi, and despite an elevated CRP, this cause seemed unlikely. The other CBC lineages, mainly other leukocytes and erythrocytes, were normal in number and form, and no immature forms were apparent in peripheral blood, rendering hematological neoplasm less likely. During this differential diagnosis path, he began complaining of new-onset asthenia and anorexia, with no other symptoms. The eosinophilia maintained its rising profile, eventually reaching the value of 41,933 eosinophils/µL. A full-body computed tomography (CT) scan was performed in search of solid tumors, revealing a left peri-hilar mass of 65 × 42 mm, obliterating the inferior bronchus (Figures 2, 3). It was hidden behind the cardiac silhouette, the reason why it was never apparent on the chest X-ray.

**Figure 4:**
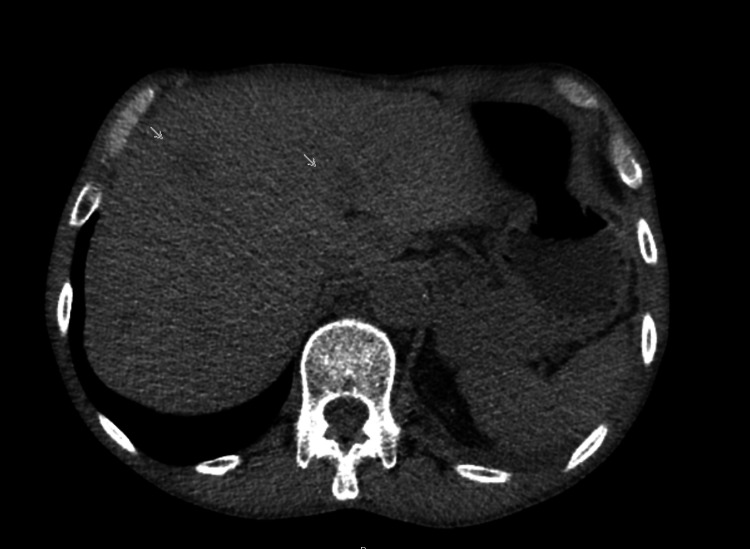
Liver metastasis During the staging of the carcinoma, multiple liver metastases were apparent in the abdominal CT scan; two of the largest ones are marked with gray arrows.

**Figure 5:**
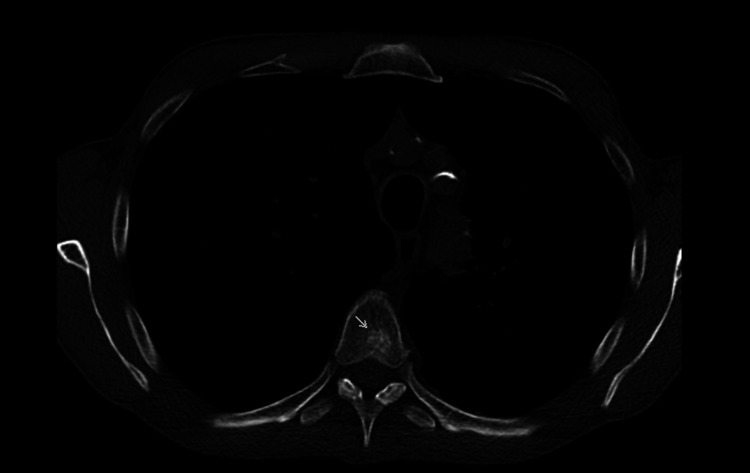
Vertebrae metastasis The staging also intended to search for eventual bone involvement and found a metastasis in the fifth thoracic vertebra, which was marked with a gray arrow.

In the abdominal segments of the CT scan, multiple nodules were visible on the liver (Figure 4), and a nodular sclerotic area of 11 mm was visible along the D5 vertebra (Figure 5).

**Figure 6:**
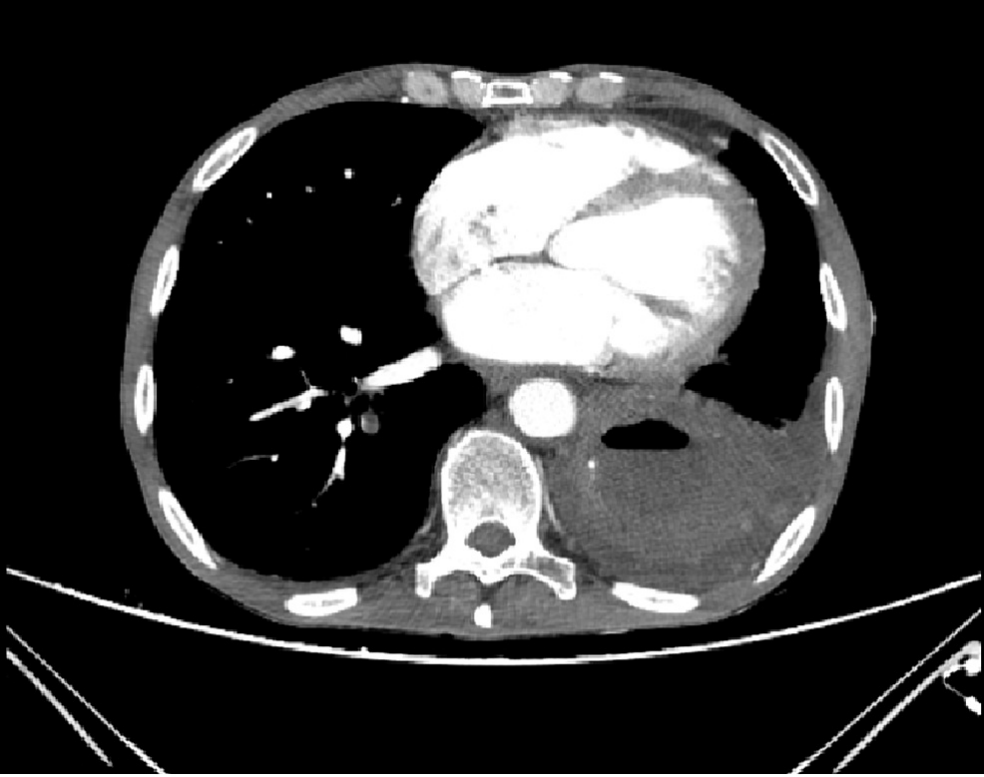
Lung abscess The patient was readmitted to the hospital with high fever and dyspnea caused by superinfection of the neoplasm region, with the formation of the lung abscess shown in the figure, with an evident gas-fluid level.

A pulmonary biopsy was not performed due to the centrality of the lesion and the risk of iatrogenic pneumothorax. The liver nodule was biopsied instead, revealing a hepatic metastasis of non-small lung cell carcinoma, and the diagnosis of stage IV small cell lung carcinoma was made. When a treatment course was being programmed, the patient started complaining of thoracic pain and was readmitted to the infirmary with the diagnosis of pulmonary thromboembolism. He then began having fever and a rise in serum inflammatory markers, with the CT scan showing a pulmonary abscess in the lower left lobe (Figure 6), in line with the recently discovered left bronchi obstruction by the tumor.

He was started on broad-spectrum antibiotics, and the infectious complication precluded the onset of chemotherapy. The respiratory failure aggravated fast despite all measures taken, and it was decided to privilege comfort measures. He came to die a mere two weeks later.

## Discussion

Eosinophils were first described by Paul Ehrlich in the 19th century as cells with granules that had an affinity for the then recently created eosin dye [3]. Several decades later, eosinophils were found to be mainly tissue cells with biological functions such as phagocytosis, antigen presentation, hemostasis activation, and direct cell killing mediated by the cytotoxic contents of the granules they possess. They are produced in the bone marrow under the influence of circulating and local cytokines, mainly interleukin (IL)-5, IL-3, and granulocyte-macrophage colony-stimulating factor (GM-CSF). An accumulation of eosinophils in peripheral tissues, essentially due to the aforementioned capacity of direct cytotoxicity and the production of inflammatory cytokines, can be responsible for organ damage [4,5]. The organ damage associated with eosinophilia can be diverse and can virtually implicate any organ. It most commonly affects the skin, lungs, and gastrointestinal tract, as well as the heart and central nervous system [6]. Independent of the absolute count of eosinophils and the presence or absence of organ damage, eosinophilia can be divided into two groups. Primary or monoclonal eosinophilia occurs when the excess of eosinophils is caused by the proliferation of a bone marrow clone, such as in myeloid and lymphoid neoplasms, systemic mastocytosis, and primary hypereosinophilic syndrome. Secondary or polyclonal eosinophilia occurs when there is a stimulus responsible for the hyperproduction of eosinophils, which occurs in parasitic infections, allergic reactions, exposure to drugs, solid tumors, and several rheumatological diseases [7].

There are very few cases published in the literature portraying this degree of extreme eosinophilia [8]. In the case described above, the initial presentation was in the form of acute heart failure with non-ST-segment elevation myocardial infarction. The TTE and cMRI both suggested the diagnosis of nonischemic cardiomyopathy. This finding, in a patient with a previous history of alcohol consumption (which is commonly underestimated), made us assume to be in the context of alcoholic cardiomyopathy, eventually with superimposed ischemic defects. We can only presume the cause of the cardiomyopathy to have been related to eosinophilia since an endomyocardial biopsy was not performed and the patient had documented coronary atherosclerosis and alcohol consumption, and as such, various etiologies are concurrent and could even be synergistic. We found several cases of eosinophilic cardiomyopathy described in the literature [9-11]. Eosinophil infiltration and tissue damage can virtually occur in any cardiac structure, leading to correspondent damage. It is assumed that heart damage mediated by hypereosinophilia is multifactorial and consists of various mechanisms: 1) the eosinophil myocardial infiltration and the release of cytotoxic products can directly damage the myocardium and provoke a form of necrotic myocarditis, 2) the inflammatory substances secreted by the locally deposited eosinophils stimulate fibrosis and consequently a form of restrictive cardiomyopathy, 3) endocardial damage can result in the formation of intracardiac thrombus and systemic or coronary embolization, 4) the infiltration in the coronary arteries themselves can damage the vessel and cause a myocardial infarction, and 5) valvular insufficiency can occur with the deposition of eosinophils in valvular leaflets [12,13]. Clinically speaking, various phenotypes can take place, from acute fatal myocarditis to chronic heart failure passing through acute myocardial infarction and valvular regurgitation. There is some disagreement as to whether the severity of eosinophilia is related to its clinical manifestations [14]. There have been cases described of severe eosinophilia without any organ damage or even symptoms, as well as mild or absent eosinophilia with fatal complications related to eosinophil organ infiltration. Our patient had only mild eosinophilia at the beginning and, despite the progressive worsening of cell counts, reaching the enormity of more than 40,000 eosinophils/µL, was persistently asymptomatic if we credit the major proportion of the heart failure to alcoholic and ischemic disease. On the other hand, there is little debate as to whether the etiology has any role in the degree of eosinophilia; there seems to be a clear relationship between severe eosinophilia (more than 5,000 eosinophils/µL) and malignant causes [15]. Our patient had, indeed, a solid tumor that was found after an extensive search, since it was not apparent throughout the first diagnostic pursuit and there were not any symptoms leading to the suspicion of a particular tumor.

## Conclusions

In conclusion, this case emphasizes two main aspects. Firstly, it is important to maintain a high degree of suspicion when facing severe analytical disturbances, even in apparently asymptomatic patients. Eosinophilia is a common finding in patients, usually associated with allergies or drug reactions. However, in this case, the persistence of the elevated counts (with no clear etiology) and their exuberance made us think of possible alternative diagnoses, which ultimately led to the diagnosis of pulmonary adenocarcinoma. Secondly, uncommon causes of acute heart failure should not be forgotten, even in patients with cardiovascular or other risk factors. Our patient had an alcohol abuse problem and coronary disease, both of which could justify the acute heart failure. Nonetheless, due to the high prevalence of both in the general population, they can be present without being the culprit of heart failure. An attentive diagnostic pursuit of uncommon etiologies can reveal unexpected diagnoses.
